# Using Facebook for Qualitative Research: A Brief Primer

**DOI:** 10.2196/13544

**Published:** 2019-08-13

**Authors:** Daschel Franz, Heather Elizabeth Marsh, Jason I Chen, Alan R Teo

**Affiliations:** 1 Center to Improve Veteran Involvement in Care Veterans Affairs Portland Health Care System Department of Veterans Affairs Portland, OR United States; 2 Department of Psychiatry Oregon Health & Science University Portland, OR United States; 3 School of Public Health Oregon Health & Science University and Portland State University Portland, OR United States

## Abstract

As Facebook continues to grow its number of active users, the potential to harness data generated by Facebook users also grows. As much of Facebook users’ activity consists of creating (and commenting on) written posts, the potential use of text data for research is enormous. However, conducting a content analysis of text from Facebook users requires adaptation of research methods used for more traditional sources of qualitative data. Furthermore, best practice guidelines to assist researchers interested in conducting qualitative studies using data derived from Facebook are lacking. The purpose of this primer was to identify opportunities, as well as potential pitfalls, of conducting qualitative research with Facebook users and their activity on Facebook and provide potential options to address each of these issues. We begin with an overview of information obtained from a literature review of 23 studies published between 2011 and 2018 and our own research experience to summarize current approaches to conducting qualitative health research using data obtained from Facebook users. We then identify potential strategies to address limitations related to current approaches and propose 5 key considerations for the collection, organization, and analysis of text data from Facebook. Finally, we consider ethical issues around the use and protection of Facebook data obtained from research participants. In this primer, we have identified several key considerations that should aid health researchers in the planning and execution of qualitative studies involving content analysis of text data from Facebook users.

## Introduction

Social media platforms provide an information-rich opportunity to reach diverse populations that would otherwise be difficult to identify. Facebook, in particular, is the most dominant player in the social media landscape. Over the past decade, the number of active Facebook users has grown from 145 million in 2008 to more than 1.2 billion in 2018 [1,2]. As of 2018, approximately two-thirds of US adults use Facebook [3]. In addition, about 75% of Facebook users visit the site at least once per day and spend upward of 50 min daily on Facebook [3,4], where they get entertainment, read news, communicate with friends and family, and exchange social support [5].

As a significant portion of individuals’ social lives is conducted (and hence displayed and recorded) on Facebook, it is a potentially rich source of qualitative data for researchers [6]. Numerous studies ranging in topic from psychopathology [7,8] and chronic physical illnesses (eg, cancer or diabetes) [9,10] to substance use [11,12] have incorporated data from Facebook, recruited from and included Facebook users as study participants [13,14], or conducted behavioral interventions on the Facebook platform [12]. Despite the rising number of studies on Facebook, relatively little is understood about how qualitative data from Facebook users can best be captured and used for health research purposes. Individual and group interviewing, focus groups, individual and group ethnographic interviewing, and observational data are among the most common methods used to traditionally collect qualitative data [15-17]. These sources of qualitative data naturally allow researchers to unpack deep meaning within a select group of people [18], probe for underlying values, beliefs, and assumptions [19], and obtain more nuanced or novel information than that derived from other methods such as close-ended survey questions [19]. However, because of the nature of Facebook data, qualitative research methods may require additional adaptation to best capture the visual, virtual, and textual interactions on social media with accuracy [20].

In this primer, we explore the opportunities, as well as potential pitfalls, of conducting qualitative research with Facebook users and their activity on Facebook. Our focus here is purposefully narrow. We limit our approach to content analysis and user-generated text related to health topics on Facebook. We begin with an overview of the forms of qualitative data and data analysis best suited to the Facebook environment, focusing on text data generated by Facebook users. Then, we consider gaps in current qualitative methods based on the existing published literature. Finally, we present 5 key issues that must be addressed in a successive manner when conducting qualitative content analyses of health-related topics involving Facebook data, and we offer potential options to address each of these issues.

## Overview of Using Qualitative Data on Facebook

Data obtained from Facebook users offer substantial opportunities for qualitative researchers. As described in Table 1, user-generated videos, images, reactions, and text are a rich source of qualitative data on Facebook. For the purpose of this paper, we focused on user-generated textual data. There are 3 primary types of user-generated textual data on Facebook:

1. *Posts:* A post is written by a Facebook user, and that post then appears on another Facebook user’s timeline. A *status update* is a common type of post in the Facebook environment, which will appear in the *news feed* of a user’s Facebook friends. A news feed is a list of updates from a user’s Facebook friends that is intended to provide the user a quick update on what their Facebook friends have been doing on Facebook.
2. *Comments*: A comment is a response to a Facebook post or a response to another comment itself.
3. *Messages:* A message is privately sent from one user to another Facebook user, typically a Facebook friend. A message does not appear on a user’s Facebook timeline or in their news feed.

All 3 of these types of user-generated text on Facebook may be accompanied by image(s), video(s), and/or emoticon(s). An emoticon, or *emoji*, is a graphic facial expression that can appear embedded in text communication on Facebook and is primarily used to provide emotional information that would otherwise only be found in traditional face-to-face interactions (eg, tone of voice) [21].

Social media qualitative research methods can be described in 3 ways: active analysis, passive analysis, and research self-identification [22]. *Active analysis* on Facebook involves the participation of research members in communication with Facebook participants. For instance, Cheung et al [11] created a study Facebook group and invited participants to join. The research team member serving as the Facebook group moderator actively participated in generating content (ie, posts and comments) that aimed to stimulate engagement with study participants. *Passive analysis* on Facebook involves the study of information patterns observed on Facebook or the interactions between users in existing Facebook groups. For example, Kent et al [13] investigated public attitudes about obesity and cancer by performing a keyword search on Facebook to identify relational themes, grammatical elements, and valence of the sentiments contained in Facebook posts and associated comments. Finally, *research self-identification* is when researchers use Facebook as a research recruitment tool to gather participants for Web-based interviews, focus groups, or surveys. For example, Pedersen et al [14] designed 3 different sets of study advertisements that appeared on approximately 3.6 million targeted Facebook users’ news feed. By clicking on the study advertisements, Facebook users were redirected to a study survey and were given the option to participate in the study.

To determine current approaches to the use of qualitative data on Facebook, we performed a literature search in April 2018 for papers that used qualitative methods to analyze user-generated Facebook text related to health topics (ie, any acute or chronic disease including substance abuse disorders). Our review identified 23 studies published between 2011 and 2018. The majority of these studies extracted data from public Facebook pages or groups [7-11,13,23-37]. Of 23 studies, 18 used passive analysis [7-10,13,23-28,30-36], 5 used active analysis [11,12,29,37,38], and none used research self-identification. Among the passive analysis studies, the number of posts, comments, and groups or pages analyzed ranged from 25 to 500, 233 to 15,972, and 1 to 840, respectively. In addition, among the active analysis studies, the number of posts analyzed and participants included ranged from 6 to 469 and 79 to 160 participants, respectively. Nearly all studies used a process of manual coding, although 1 study used machine learning techniques [37]. A wide range of health issues was examined from breast cancer to smoking cessation. Further descriptive characteristics can be found in Multimedia Appendix 1.

**Table 1:** Potential sources of data for qualitative data analysis within Facebook.

Filters	Data included
Timeline	User-generated and user-directed posts, comments, reactions, shares, photos, videos, tagged posts and photos, and when the participant added someone as a friend. Displays public data
Activity log	User-generated and user-directed posts, comments, reactions, shares, photos, videos, tagged posts and photos, pages liked, and when the participant added someone as a friend. Displays public and private data
Posts	Posts generated by the user
Posts tagged in	Posts where other users tag the user
Others’ posts to your timeline	Posts that others generate on the user’s timeline
Hidden from timeline	A privacy setting that limits who can see posts on a participant’s timeline
Photos and videos	Photos and videos that the user posts, uploads, or is tagged in
Likes and reactions	Likes and reactions generated by the user
Comments	Comments generated by the user
Articles you have read	Articles read by the user
Notes	User-generated full-length posts without limited character length and can include tagging and pictures
Videos you have watched	Videos watched by the user
Following	A list of pages the user follows
Groups	A list of groups the user is a member of
Search history	Content the user searches on Facebook

## Gaps in Current Qualitative Approaches

Our review identified a number of limitations within the existing literature. First, most studies did not provide detailed descriptions of their methods [39,40]. In particular, description of data extraction methods was frequently missing [7,11,13,23,25,27-31,33,34,37]. Furthermore, there are few existing resources that offer guidance for researchers seeking to use Facebook for health-related topics. Lack of methodological descriptions and advice in the literature pose as barriers to researchers trying to replicate study results or apply the same methods in pursuit of novel research questions in the health domain. Second, none of the studies analyzed bidirectional interactions among participants and other Facebook users. Bidirectional interactions are social exchanges of user-generated and received text between Facebook users. Received text is text directed to a Facebook user, such as a friend’s comment to that Facebook user’s post (hereafter, user-directed text). These interactions are commonly displayed as a chain of communication on a user’s timeline or news feed that exemplify how individuals use and interact with others on Facebook. By collecting only user-generated text or user-directed text on Facebook, studies are only capturing one side of Facebook user’s interactions with other Facebook members. However, collecting bidirectional interactions provides more context of social exchanges on Facebook, which can assist in more meaningful interpretations of the data. Therefore, it is important to establish methods for researchers seeking to capture this type of information. Third, most studies that included either manual or machine-coding techniques lacked familiarization methods before coding [8,11-13,24-27,29-38]. Familiarization methods include researchers immersing themselves with the data before coding by actively reading the data to understand the depth and context of the content [41]. To conduct rigorous and trustworthy thematic analyses, it is vital to read through the entire dataset at least once before coding [41,42].

Owing to these limitations, in this paper, we identify and discuss 5 key issues in the process of conducting qualitative research using data obtained from Facebook. These issues are summarized in Textbox 1 and described in detail below. In addition, we use our own experience from a recent research project to illustrate 1 potential approach to handle each of these issues. Our experience derives from a study in which we used Facebook advertisements to recruit a sample of military veterans [43]. Study participants completed a Web-based survey about their psychiatric symptoms and social support, and a subgroup was invited to participate in an additional in-person study visit in which they provided access to some of their Facebook data. For qualitative analysis in this project using Facebook data, we used content analysis, which, for our study, was a more directed approach that allowed us to begin by identifying key concepts and variables as initial coding categories.

**Key considerations for future studies using qualitative approaches for social media data.**

**Step 1. What kind of Facebook user will be included in the study?**

- The method of recruitment of Facebook users will affect participants’ characteristics and generalizability of results.
- The degree of activity on Facebook by a study subject will impact the amount of data available for analysis.

**Step 2. What Facebook data will be analyzed?**

- Facebook contains a combination of public and private information about individual users.
- Filters can be used to select desired variables and data about Facebook users.
- It is helpful to predetermine a period of Facebook use to be included in data analysis.

**Step 3. How will the Facebook data be obtained?**

- Options include partnering with Facebook, collecting publicly available data, creating a research study–specific Facebook page or group, or downloading participants’ Facebook data.
- Each option has pros and cons related to the complexity of the process and comprehensiveness of data obtained.

**Step 4. How will the Facebook data be analyzed?**

- Depending on the size of the dataset, researchers may prefer a manual versus more automated approach to coding and data analysis.
- Qualitative data analysis and other software can assist with the data analysis.
- Consider the model of qualitative analysis used in the study.

**Step 5. How will participant’s Facebook data be protected?**

- The Connected and Open Research Ethics is a Web-based resource [44] to help navigate ethical issues around social media research.
- Common ethical issues include the following: who will informed consent be obtained from, how will data of research subjects be kept secure, and how will the privacy of research subjects be maintained.

## Step 1: What Kind of Facebook User Will Be Included in the Study?

In deciding what kind of Facebook user will be included in the study, it is important to consider how participants will be recruited.

For studies that involve delivery of an intervention through Facebook (ie, active analysis), the platform offers 2 main features that researchers can use to recruit and maintain participants: Facebook pages and Facebook groups. Facebook pages are public, whereas Facebook groups can be public, or private or secret. In public Facebook groups, only invited members can see content. However, in secret Facebook groups, only invited members can see content, and the group is hidden—it cannot be searched for, or found, using the Facebook search engine [45]. Facebook pages and all Facebook groups can be created to recruit and conduct an intervention. In addition, researchers can access existing public Facebook pages and groups comprising current members to collect data. However, these pages and groups cannot be tailored to a researcher’s interventions. Furthermore, Facebook advertisements can be used to target a specific population by leveraging demographic profiles available on Facebook. Furthermore, Facebook advertisements can use additional information (eg, interests) added by a user to their profile. Some studies recruit both current Facebook users and other participants who are willing to open a Facebook account for the study [45]. In addition, it is important to consider the degree to which participants are regularly and actively using Facebook. Regular users will tend to have a richer record of their Facebook activity. That said, not all users of Facebook actively engage in behaviors that create a record of interaction on Facebook (eg, posting and commenting) [46]. Facebook users can be categorized into 2 types of users based on the frequency of engaging in these behaviors: active users and passive users. Active users contribute to Facebook interactions by posting and commenting frequently. Passive users tend to observe Facebook interactions and not actively contribute. For active analysis studies, both active and passive users can be considered for recruitment. Interventionists may consider designing posts to initiate interactions among participants, especially from passive users.

In addition, studies intending to observe Facebook user’s interactions with other users (ie, passive analysis) can use 2 public group features available on Facebook: Facebook pages and public Facebook groups. As these pages and groups are public, researchers are able to openly view all Facebook data without restrictions. As a result, researchers can search for an existing public page or group related to a health topic of interest and then collect the data presented within the page or group. Data found in public Facebook pages and groups can be from both active and passive users. Typically, there is a direct relationship between the number of members part of a Facebook page or group and the amount of data available. One drawback about using public Facebook pages and groups is that the pages and groups about a health topic of interest must already exist. Alternatively, passive analysis studies can recruit Facebook participants individually through Facebook advertisements. An advantage of this approach is the ability to continue an advertising campaign until enough participants and data are collected, whereas a disadvantage of it is the requirement for a nontrivial advertising budget.

Paid advertisements on Facebook are also useful for studies seeking to recruit participants from Facebook to participate in interviews, focus groups, surveys, or other research activities (ie, research self-identification). Facebook advertisements can be used to target particular users using the methods described above. Facebook users can be directed to a study website when they click on the advertisement, which then can further describe the study and include Web-based informed consent. Furthermore, Facebook advertisements can record user actions such as advertisement clicks (ie, number of times the advertisement was clicked on) and comments on the post containing the advertisement.

Finally, as with other Web-based studies in which in-person contact with a study participant does not occur, exclusion criteria should be carefully considered to reduce misrepresentation of participants and potentially counterfeit responders (ie, responders pretending to fit a certain demographic for study compensation).

### An Applied Example

We used research self-identification methods to recruit participants through Facebook advertisements [43]. Advertisements contained a call to action to participate in a health research study. Study advertisements broadly targeted Facebook users in the United States of any age or gender who had interests relevant to military veterans. Advertisements were hosted by Facebook pages affiliated with our university. This allowed us to draw on the established base of Facebook users interested in and following our university on Facebook.

To reduce misrepresentation of participants, we excluded individuals who completed the survey in less than 5 min, had a duplicate or multiple survey responses, or incorrectly answered military-related *insider knowledge* questions [14,47]. To help ensure study subjects had enough Facebook data to analyze, we chose to collect qualitative data from participants who reported using Facebook at least once a day.

## Step 2: What Facebook Data Will Be Analyzed?

In deciding what Facebook data will be analyzed, it is critical to determine the setting in which the data will be collected. For active or passive analysis studies collecting data from public, private, or secret Facebook groups or pages, it is important to consider downloading individual Facebook user’s profile information in addition to the information exchanged in groups or pages. A Facebook user’s profile information shows how the user interacts in multiple Facebook settings compared with a singular setting (ie, a Facebook page or group). Therefore, collecting and analyzing data from a user’s Facebook profile provides more context to how they interact, whom they interact with, and in which environments (ie, public or private) they are more active. Understanding how research participants interact on Facebook can be used to supplement the context of the responses and inform future intervention processes.

In addition, given how expansive the amount of Facebook data can be, even just from a single Facebook user, it is vital to determine the scope of data that will be analyzed. As described in Table 1, Facebook features, such as *Filters*, allow data to be viewed in already separated Facebook variables such as user-generated data (ie, notes, posts tagged in, and timeline review). These filters can be manipulated to display specific data of interest. Although filters can help find user-generated and user-directed data, it is important to also capture these same data in the timeline. The timeline shows how Facebook users are interacting, which helps provide context when analyzing the data.

Furthermore, it is also important to determine how long it takes to collect the Facebook data. Data collection time is dependent on how active the Facebook user is and, for pages or groups, how many users are part of a page or group. These factors can impact additional study procedures (eg, interviews) at the time of the Facebook data collection period.

### Our Experience and Applied Example

In our study, we sought to capture all our veteran participants’ written social interactions on Facebook. We did this by collecting user-generated and user-directed comments, status updates, and posts from the activity log and the timeline. The timeline was also included as it contains data from both public and private settings on Facebook. By collecting both user-generated and user-directed data, we were able to capture bidirectional interactions between study participants and other Facebook users within their social network.

In addition, data were collected over a 4-week period around the time of the participants’ survey completion. We decided to collect participant’s Facebook data at the time of the in-person interview so that a research member could be physically present to assist a participant in the process of downloading his or her Facebook activity. After informed consent, the initial 10 min of the session were used to collect the participant’s Facebook activity information, which was sufficient to collect users’ Facebook data, ranging up to approximately 70 user-generated posts.

## Step 3: How Will the Facebook Data Be Obtained?

### Option 1: Partner With Facebook

Facebook data can be obtained through a research partnership with Facebook. Kramer et al [48], supported by Facebook resources, collected posts and manipulated news feeds of 689,003 Facebook users over a 20-year period. Burke and Kraut [49], led by a Facebook researcher, collected user-directed comments, private messages, timeline posts, likes, and pokes, as well as user information such as number of profiles viewed, news feed stories clicked on, and photos viewed from 10,557 Facebook users. Some advantages of partnering with Facebook are that studies can have access to massive amounts of data including Facebook variables that are not shared with users or third parties [50]. In addition, one can leverage Facebook resources (ie, data processing systems) to track how much people are discussing specific topics of interest and the subsequent opinions of those topics expressed in everyday conversation. Such Facebook resources efficiently gather large-scale data in which data are retrieved almost instantaneously. However, a challenge of partnering with Facebook is meeting their *collaborative requirements*, such as finding a Facebook sponsor to lead the research effort, and the faculty principal investigator’s institution paying up to 40% of overhead costs for a hosted researcher [51]. Therefore, this process can be resource intensive in terms of both time and financial investment by the partner researcher.

### Option 2: Publicly Available Data

Active and passive analysis studies can obtain Facebook data through public Facebook pages and groups. There are several studies using extraction methods such as manual extraction (eg, copying and pasting data into a spreadsheet) or contracting through external models and third-party services for manual extraction. Abramson et al [9] copied and pasted each public timeline post from the Breast Cancer Organization page into a spreadsheet with the corresponding responses. Eghdam et al [8] used Netvizz version 1.25, a data collection software created by Facebook, to collect anonymous data from public Facebook groups. Kent et al [13] used a Web-crawling service that mined publicly available posts and comments from Facebook using keywords related to obesity. Furthermore, Kosinski et al [50] provide Pennebaker’s Linguistic Inquiry and Word Count (LIWC), and the Apply Magic Sauce, a website developed by the University of Cambridge psychometrics center, [52] as an additional resource for data collection. An advantage of using public data is that there are a lot of data for a range of health topics, and informed consent by the participant is not required. However, the challenge of using data shared publicly could be biased because of social desirability influences and other censoring by a given participant. Studies suggest that both privacy concerns and the user’s audience can impact self-disclosure on Facebook, especially when it comes to sharing health information [53-57]. Eysenbach and Till [22] recommend working with group moderators to develop an adequate plan for informing group members of the use of their data. Although they identify obtaining permission from the group moderator as insufficient on its own, group moderators have greater knowledge of their group members and may be able to provide important information on how to best obtain consent for use of data.

### Option 3: Create and Monitor a Facebook Page or Group

In addition, for active analysis studies, Facebook data can be obtained by creating and monitoring a Facebook page or group. Beullens and Schepers [12] collected 2575 pictures and 92 status updates by creating a study Facebook profile and sending friend requests, including a study overview message, to 166 college students. Tower et al [38] collected post information by creating a Facebook group and inviting 198 nursing students to join the group through email. The invitation advised the group to post information related to their study. A faculty member initiated discussion in the Facebook group. The text and associated attributes were downloaded onto a spreadsheet. An advantage of creating and monitoring a Facebook page or group is that it allows a research team to customize a group specific to a particular health topic. Subsequently, targeted individuals can be invited to this page or group and be presented a set of specific questions/instructions to stimulate participant engagement. In addition, only group settings can be made private, which can create a more secure environment for participants to disclose personal information. However, a disadvantage of private groups is that there is a permanent setting that organizes user-directed posts such that the most recent interactions appear at the top of the group feed versus a chronological ordering of the post [45]. As a result, posts containing important content may be pushed to the bottom of the group feed because of frequent posting in the groups, thereby making it difficult for participants to find information posted by the groups interventionists [45]. In addition, although Facebook groups can be private or secret, they are still not the Facebook user’s *natural environment* —that is, the social network comprising Facebook friends the user normally interacts with. Therefore, Facebook users recruited into an intervention conducted in a private or secret group may behave differently in groups created by researchers, especially when they know they are being observed by researchers [58].

### Option 4: Private Messages

Furthermore, for active analysis studies, Facebook data can be obtained by asking participants to copy and paste user-generated Facebook text (eg, text from timeline posts or private messages) and provide it to a research team member through a Web-based portal or through private messaging to a Facebook account created by the research team. Bazarova et al [37] collected 474 most recent status updates, timeline posts, and private messages by inviting 79 participants to copy and paste their data into a Web survey. An advantage of having users provide their Facebook data through the private messaging feature or a Web-based portal is that it creates a secure environment in which participants’ Facebook data can be kept confidential from other Facebook users or study participants. However, one disadvantage of this particular method is that researchers would neither be able to observe passive interactions among a particular group of Facebook users nor observe interactions as a result of a proposed set of questions/instructions regarding health-related topics.

### Our Experience and Applied Example

**Figure 1:**
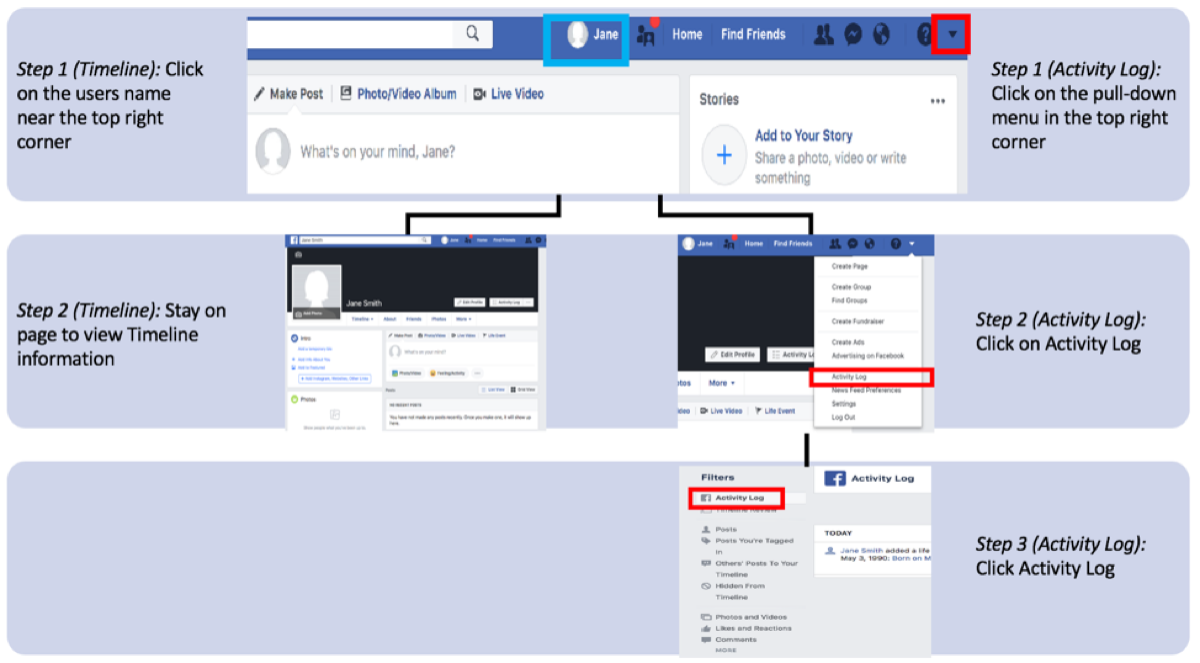
Steps to access the timeline (eg, blue square) and activity log (eg, red squares) on Facebook.

A fourth option, applicable to active and passive analyses and some research self-identification studies, is directly downloading participants’ Facebook data during an in-person study visit. We chose this option because it was the only one that allowed us to download individual’s Facebook profiles without establishing a partnership with Facebook. For instance, in our own study, we obtained Facebook data by downloading participants’ Facebook activity information. During the in-person interview, users’ Facebook activity log and timeline data were collected separately by study staff using the following steps: (1) ask participants to login to their Facebook account, (2) follow the steps described in Figure 1, (3) scroll *backward* on the selected page chronologically until 1-month period before the date of the survey; (4) save as an HTML file on OHSU Box (a cloud-based data storage service that complies with local security and regulatory policies), (5) open saved file with Safari to view extracted data, (6) log participants out, and (7) ensure that no username or password information was retained by making sure user login information was not saved by the browser. We noted some advantages of downloading participants’ Facebook profile information, such as a participants’ Facebook profile can provide insight to how individuals interact, who they interact with, and what environment (ie, public and private) they are more active in. This helped us understand how study participants interacted on Facebook. However, a challenge of downloading participants’ Facebook profile information is that it requires participant consent, and it can be more difficult to collect massive quantities of private data because of the length of the collection period.

## Step 4: How Will the Facebook Data Be Analyzed?

Qualitative Facebook data are commonly analyzed using methods such as content analysis to assess a wide range of qualitative data or else constant comparison to identify themes [6]. In deciding how qualitative Facebook data will be analyzed, it is important to consider the quantity of the data as well as the qualitative approach being used. For active and passive analysis studies using larger datasets, it is preferable to analyze data using software programs. AlQarni et al [34] analyzed 1551 posts using predetermined themes, and further inductive codes were used to independently extract and analyze the Facebook posts to determine major content themes. Thematic analysis was performed using NVivo, a qualitative software used to code, store, and potentially exchange data with SPSS for further statistical analysis. Kramer et al [48] used LIWC (2007) software to analyze 689,003 posts to determine if the valence of the posts was positive or negative. Keller et al [32] used ATLAS.ti, a qualitative software used to code data, to code 1614 comments for major and minor themes. It is important to note that ATLAS.ti can be used to code HTML files of individual’s Facebook downloads; however, this has not been done in social media qualitative research studies [59]. Instead, ATLAS.ti has been traditionally used to code Microsoft Word documents of transcribed interviews.

### Our Experience and Applied Example

As our study contained a relatively small dataset (23 subjects with 201 posts and 424 comments), we opted to analyze data manually. User-generated text from status updates, posts, and comments and user-directed text from posts and comments from the HTML files were copied and pasted into an Excel spreadsheet and analyzed for markers of social support. Our codebook contained 3 different types of social support that have previously been described in the literature (emotional, instrumental, and informational) and a fourth category for other evidence of social support (eg, “Wow, that’s a great joke”). In addition, we coded the valence of user-directed social support as positive, negative, or neutral. Before coding, each coder read over the entire dataset to familiarize themselves with the content of the data. The familiarization process helped lead to more meaningful interpretations of the data because we were able to easily provide context to each piece of text we coded. As is common in qualitative research, after an initial training period, 2 coders independently coded participants’ data. Furthermore, each coder created a memo describing their experiences during the coding process. This highlighted the challenges and successes of the coding process, which guided conversations around any discrepancies. In addition, the memo process brought awareness to potential challenges of coding text on social media, which can be addressed early on for future social media qualitative work. Furthermore, the memo process also identified general themes that were prevalent in the data.

## Step 5: How Will Participants’ Facebook Data Be Protected?

It is important to highlight that Facebook research raises several ethical questions. Owing to the nature of studying Facebook communities, researchers can potentially violate the privacy rights of Facebook users. Facebook users that are members of public Facebook pages or groups do not expect to become research subjects nor do the Facebook friends of study participants (ie, *nonparticipants*). The boundary between private and public Facebook data may sometimes be unclear. The majority of Facebook users are aware that their data may not be private [22], especially in a public setting on Facebook. However, the literature regarding social media users’ comprehension of privacy literacy is limited [60]. As a result, researchers should ensure that informed consent language is clear regarding how a participant’s Web-based data will be used. Pilot testing of informed consent language may help ensure that the information presented is easily comprehensible for a broad range of populations. Regardless, it is important to maintain the safety and anonymity of individuals’ Facebook information whether or not they are a research participant.

In addition, it is important to note the potential ethical dilemmas associated with establishing a research partnership with Facebook. Facebook is a powerful company with a rich source of data; however, Facebook has received public scrutiny because of their misuse of their users’ Facebook data. Therefore, the responsibly is placed on the research teams to ensure that Facebook users’ data are obtained ethically and protected. Arigo et al [61] recommend including research team members who are well versed with Facebook’s cooperate terms and conditions and privacy policies. It is strongly encouraged that research teams are knowledgeable of the peculiarities of Facebook before establishing a partnership to assist in the development of research methodological procedures regarding data collection and privacy.

As each institutional review board (IRB) will vary in its familiarity with social media research, we recommend closely consulting with professional and independent organizations (eg, Association of Internet Researchers Ethics Working Group Guidelines, The National Committee for Research Ethics, and The Humanities Research Ethics Guidelines for Internet Research) as well as Web-based resources such as the Connected and Open Research Ethics (CORE). CORE can provide assistance in how to address potential ethical issues for researchers and IRBs interested in social media research. Common ethical questions that have been raised on CORE include the following: (1) Who will informed consent be obtained from–is informed consent required for *nonparticipants* on a research subject’s account?; (2) How will data from research subjects be kept secure on the social media platform?; and (3) How will the privacy of research subjects be maintained? CORE has created a collaborative platform where researchers can exchange expertise and questions pertaining to social media research. Features such as the *Resource Library*, *Q&A Forum*, and the *CORE Network* provide scientists access to IRB-approved research protocols and consent forms and allow researchers to discuss collaboratively ethical design or potential social media strategies [44].

### Our Experience and Applied Example

In our study, participants interested in an optional, in-person interview provided contact information with which study staff used to arrange the study visit. For individuals who were unable to come in-person, we conducted interviews through phone but did not download their Facebook data. Overall, 2 separate informed consents were obtained, once online for those completing the survey and again in-person for those sharing their Facebook data. During the informed process for those sharing their Facebook data, participants were informed that their timeline and activity log would be collected to observe their online social interactions and Facebook usage. In addition, participants were informed that their Facebook data would be labeled with a unique code to protect their identity. All study procedures were approved by the IRB of Oregon Health & Science University.

## Limitations

There are several limitations to this study. First, this study represents 1 proposed framework. Additional validation of this framework among other experts would be a helpful next step. Second, the scope of the study is limited. We primarily focused on content analysis of user-generated Facebook text related to health topics using a content analysis approach to qualitative analysis. Studies that intend to use other models of qualitative analysis may require somewhat different approaches to the use of data from Facebook. Nontext qualitative data from Facebook (eg, images, videos, and emoticons) also bear further examination. Third, because our key considerations are primarily directed toward health-related studies, it is unclear whether they are generalizable to other research topics that harness data from Facebook. Finally, our applied example did not address methods for collecting data from existing closed Facebook groups, although studies that did do so were identified in our literature review. Studies that involve interaction with Facebook group members require additional consideration, and future research could help elucidate this area by extending the work presented by Eysenbach and Till [22].

## Conclusions

Although there are an increasing number of studies that are using qualitative data obtained from Facebook users, there has been little published to date, summarizing the current state of this research. Our review of the literature and own experience conducting this type of research have led us to identify several key considerations for health researchers interested in conducting qualitative studies involving Facebook data. Our hope is that future research continues to refine and develop approaches to conducting research in this exciting area.

## Appendix

Multimedia Appendix 1 Summary of qualitative peer-reviewed studies using Facebook as a data source.

## Abbreviations

CORE: Connected and Open Research Ethics
IRB: institutional review board
LIWC: Linguistic Inquiry and Word Count
