# Supplementary material for: Using Facebook for Qualitative Research: A Brief Primer
Source: J Med Internet Res. 2019 Aug 13;21(8):e13544. doi: 10.2196/13544 (PMC6711038; doi:10.2196/13544)
Supplement: Multimedia Appendix 1 [file jmir_v21i8e13544_app1.docx]

| Study | Year of data collection | Sample size | Data privacy | Population | Type of analysis | Extraction | Coding process |
| --- | --- | --- | --- | --- | --- | --- | --- |
| Cheung et al^a^ [12] | N/A^b^ | 467 posts by the 82 group members | Private | Smoking cessation | Active analysis | Participants of a smoking cessation study were randomly assigned to 3 randomized controlled trial social media groups (WhatsApp, Facebook, or a control group) where they were invited to join a Web-based discussion through their assigned social media group. A moderator would participate when no participants responded to others’ posts for a day or when advice was needed. All discussion content was archived and converted to anonymous transcripts for content analysis. | Content related to social support and smoking relapse was analyzed by identifying posts regarding sharing experiences, encouragement, perceptions, knowledge, and suggestions. Each participant’s post was initially coded with one or several thematic codes. Two research members independently recoded all discussion content to maintain consistency. All discrepancies would be discussed during 2 panel sessions until a consensus was met. Revised codes were independently coded by the first author and a trained research member to determine interrater reliability. The definitions of nodes below 0.6 were discussed and redefined in the third panel discussion. The first author either coded again with a revised definition or reallocated those posts to other codes. |
| Kent et al [14] | 2012 | 291 posts | Public | Obesity | Passive analysis | The original dataset was created using a Web-crawling service, which mined publicly available posts and comments from social media sites, including Facebook with keywords related to obesity. A subset of the parent dataset was generated by extracting posts and from Facebook with cancer-related keywords. | Content was analyzed using grounded theory approach. Three researchers independently double-coded the data. All discrepancies were reviewed by 2 additional coders, and final adjudication was performed by an additional reviewer. Four categories of sentiment valence were coded. Concurrent embedded design was chosen, such that a descriptive qualitative analysis was embedded within the more dominant quantitative approach and used to guide, interpret, and illustrate the patterns revealed. |
| Abramson et al [10] | 2010 | 1 Facebook page, 84 posts, and 2500 comments and replies | Public | Breast cancer | Passive analysis | Each public timeline post from the Beast Cancer Organization page was copied and pasted into a spreadsheet with the corresponding responses. | Content was analyzed for distinct themes, concepts, and its primary function using grounded theory approach. One researcher initiated this process by reading each post and its associated comments on the Facebook page’s timeline. The same researcher repeated this process a second time and created a list of emerging common themes, phrases, and concepts. A second researcher also repeated this process. The 2 researchers contrasted their findings, and any discrepancies in interpretation were adjudicated by a third research member. |
| Greene et al [11] | 2009 | 15 largest Facebook groups, 480 patients, 233 timeline post comments, and 457 discussion topic comments | Public | Diabetes mellitus (DM) | Passive analysis | Performed a keyword search related to DM to find Facebook groups. The 15 most recent timeline posts from the 15 largest Facebook groups and the 15 most recent discussion topics from the 10 largest public Facebook groups were identified and aggregated into a database. | Comments were analyzed using a content analysis approach. Two researchers read all Facebook posts and developed descriptive codes based on broad themes in the data. Two researchers read comments from both the timeline posts and discussion topics and developed a unified coding scheme. Each researcher independently assigned codes to the data. Any discrepancies in coding were discussed by the 2 researchers until a consensus was reached—any conflicts were adjudicated by all 3 research team members. |
| Thoren et al [24] | 2011 | 25 largest Facebook groups, 500 timeline posts, 392 comments, and 162 discussion topics posts | Public | Preterm infants | Passive analysis | Performed a keyword search and identified public Facebook groups. The groups that met inclusion criteria were extracted, including the first 20 most recent timeline posts of each group. A maximum of the 15 first comments per timeline post and the 15 most recent discussion topics were analyzed. | Two members of the research team independently classified all posts. All discrepancies were discussed by the 2 research members until a consensus was reached. |
| Roffeei et al [8] | 2013 | 2 Facebook pages, 381 posts, and 3256 comments | Public | Autism spectrum disorder | Passive analysis | Data for this study were extracted from 2 public Facebook autism support group pages: Autism Malaysia and Autism Children Club. | Content was analyzed using deductive content analysis. Data were tabulated to generate frequencies of postings and comments. Two researchers coded the data into 5 main themes and 26 subthemes. All discrepancies were discussed by the 2 research members until a consensus was reached. |
| Cole-Lewis et al [25] | 2013–2014 | 1 Facebook page, 4243 individuals, 4088 comments, and 875 posts | Public | Smoking cessation | Passive analysis | Data were collected retrospectively from Simply Measured, a social media management marketing platform for all interactions on the public Smokefree Women Facebook page. | Applied thematic analysis of the content was conducted using an inductive methodology. Researchers first independently reviewed a subset of the data for familiarization, then rereviewed to inductively identify salient themes. Themes were then cross-referenced with previous content analyses of similar topics to develop consistent terms. Once themes were finalized, 2 researchers independently coded data for 25% of the participants to determine interrater reliability. Once interrater reliability of at least 0.8 agreement was reached, each researcher coded one half of the remaining sample. |
| De la Torre-Díez et al [26] | 2011 | 216 breast cancer Facebook groups, 171 colorectal cancer Facebook groups, and 527 diabetes Facebook groups | Public | Breast and colorectal cancer, and DM | Passive analysis | Performed a keyword search for Facebook groups. Selection of moderated groups was made, and information such as name of network, number of members, interests, and website URL were collected. | Content was analyzed. After analysis, 5 main types of groups were identified. |
| Seidel, et al [27] | 2015–2017 | 1 Facebook group and 492 posts | Public | Breast imaging radiologists | Passive analysis | Used analytic programs such as Grytics and Sociograph (to extract Facebook groups’ information such as interactions, activity, and membership) to retrospectively collect and analyze public Facebook groups’ posts, comments, reactions, and members and their demographics. | A total of 493 Facebook posts were analyzed by 3 board-certified breast imagining radiologists. The radiologists agreed on 13 descriptive categories of posts after an initial qualitative review. Each post was reviewed and then categorized by 2 of the 3 radiologists. Discrepancies in categorical assignments were discussed between the 2 reviewers until a consensus was reached. |
| Struck et al [28] | 2015–2016 | 99 Facebook groups and 283,996 page likes | Public | Prostate cancer | Passive analysis | Performed a keyword search on Facebook. Publicly available information was extracted from 58 public groups and 41 private groups. | Facebook content (number of members, likes, and posts) were identified per the criteria and were manually added to the most suitable category or named *other* if it did not match existing categories. Six functional categories were defined based on a social media literature. |
| Meng et al [29] | 2016 | 840 pages and 403 posts | Public | Epilepsy | Passive analysis | Performed a keyword search on Facebook. Extracted population data from public Facebook pages using the number of *likes* by users. Extracted the most recent 50 posts from each account category. | Posts were examined using modified thematic analysis, using open and axial coding methods. The open coding method involves sorting posts into common groups based on shared themes or ideas while axial coding subsequently categorizes all open codes based on even broader overarching themes. Two research members coded the data. All discrepancies between the 2 coders were discussed until a consensus was reached. |
| Dhar et al [30] | 2016 | 350 participants, 339 posts, 2338 comments, and 6274 reactions | Public | Liver transplants | Active analysis | Created a Liver Transplant Facebook support page to be used as a virtual community forum. Data (ie, user demographics and group activity) were compiled and reviewed. Content of posts and comments were downloaded. | Facebook posts and comments were analyzed using a qualitative thematic analysis approach. Data were independently reviewed by 2 research team members. All posts were read and categorized using a coding schema. All discrepancies were discussed until a consensus was reached. |
| Ahmed et al [31] | 2015 | 1257 timeline posts with total of 31,636 likes and 15,972 comments | Public | Dialysis | Passive analysis | Performed a keyword search on Facebook. Selected *Dialysis Discussion Uncensored* public Facebook group and collected all posts in English. Posts were manually copied from Facebook and pasted into an Microsoft Excel file. Corresponding likes, comments, and replies to the posts were recorded in the same file. Attributes associated with each post (eg, time it was posted, number of replies, comments, and likes) were also collected for each post. | Content was analyzed using a qualitative thematic analysis approach. Two research members read and independently coded all posts using an adopted coding schema from Zhang et al, which consisted of 4 major categories. Any discrepancies were discussed until a consensus was reached. |
| Gonsalves et al [32] | 2016–2017 | 25 Facebook posts, 15 links to websites, 32 images, 6 videos, 17 user comments, and 25 Heart and Stroke Foundation (HSF) and user replies to user comments | Public | Cardiovascular disease (CVD) | Passive analysis | All public HSF Facebook page posts and related user comments that contained information about women’s CVD were gathered. Data items comprised 25 Facebook posts, 15 links to websites, 32 images, 6 videos, 17 user comments, and 25 HSF and user replies to user comments. | Content was analyzed using Ethnographic Content Analysis. Data were also coded by additional categories to account for new themes refined into higher order themes. A reflective segment was used to show how categories were similar to, or different from, each other and the implications for social action. Finally, women’s CVDs conveyed through images and videos were interpreted using critical descriptions. Data analysis was systematic (eg, categories were used to guide initial data coding) and reflexive regarding novel themes and higher order connections between themes. Analysis was performed by the first and second authors. |
| Eghdam et al [9] | 2014–2015 | 1 Facebook group with 1310 members generated 630 posts, 4323 comments, and 10,187 likes on posts | Public | Brain fatigue after brain injury | Passive analysis | A Facebook group for Swedish-speaking individuals with brain fatigue after brain injury was used to collect user’s posts, written comments to posts, or acknowledged *likes* to posts. Netvizz version 1.25 was used as the data collection software developed by Facebook that allows researchers to collect anonymous data from public Facebook groups. Data were stored in comma-separated value file and graph data file format. | Posts and comments were analyzed using a direct content analysis approach. Both the first and second authors began with repeated reading of all posts and comments. For social support, the posts and comments were grouped based on the initial codes and categories of social support from the literature. In addition to posts and comments, the likes were counted. Microsoft Excel was used to code the data. The first and second authors performed the analysis process. All discrepancies were discussed until a consensus was reached. |
| Keller et al [33] | 2016 | 2401 comments | Public | Smoking cessation | Passive analysis | A video posted on Facebook by NowThis (a digital news company) yielded 36,000 shares, 67,000 likes, and 2401 comments. Microsoft Excel was used to extract the 2401 public Facebook comments. | ATLAS.ti was used to code the comments that met inclusion criteria (n=1614). Three coders performed quantitative analyses based on the codes and categories. The first round of inductive coding was used to generate a codebook of themes grounded in the data. The coders then coded the data several times to categorize each comment into sentiment categories and major and minor themes. All discrepancies were agreed on based on an established consensus process. |
| Struik and Baskerville [34] | 2012–2013 | 399 posts included for analysis, 121 were original posts, whereas the remaining 278 were reply posts | Public | Smoking cessation | Passive analysis | 399 posts were collected from the public Crush the Crave Facebook page and entered into NVivo. Posts were collected until no new themes or subthemes were identified. | The framework approach was used to qualitatively analyze the Facebook posts. The first coder coded all Facebook posts. A second coder independently coded the first 51 posts to compare for consistency. After the first 51 posts were coded, a thematic framework was developed by generating major themes and subthemes. All discrepancies were discussed until a consensus was reached. |
| AlQarni et al [35] | 2010–2015 | 1551 posts | Public | DM | Passive analysis | Facebook posts written in Arabic were collected from public Facebook groups of the 22 major Arabic-speaking countries pertaining to DM. A keyword search related to DM was conducted in Arabic. A total of 7 Facebook groups met the inclusion criteria. | Predetermined themes and further inductive codes were used to independently extract and analyze the Facebook posts to determine major content themes. Thematic analysis was performed using NVivo. Numerical data were compiled in Microsoft Excel. |
| Hale et al [36] | 2012 | 20 health conditions and the top 50 pages of results for each health condition, as well as the URL and the number of likes each page had received from Facebook members | Public | Multiple health conditions | Passive analysis | The 20 most searched posts for health conditions on Google were identified and, a keyword search on Facebook using these identified conditions was performed. The top 50 public Facebook pages were recorded for each health condition, as well as the URL and the number of likes each page had received from Facebook members. A total of 20 Facebook pages that met inclusion criteria were identified. | Used existing literature to determine categorization of Facebook pages to identify each page’s purpose. Two coauthors evaluated the 20 most recent posts on each page and categorized page content into 1 of 5 types. The 2 coders conducted an initial categorization of about 90 pages to determine interrater reliability. Another subset of 90 pages was coded using a new classification scheme, which improved interrater reliability. The remaining pages were then divided and independently coded by the coauthors. All pages were aggregated for each condition, and the number of page likes was determined. |
| Beullens and Schepers [13] | N/A | A total of 160 Belgian Facebook profiles generated 2575 pictures and 92 status updates | Private | Alcohol use | Active analysis | The second author created a study Facebook profile. Through this profile, friend requests including a study overview message were sent to 166 college students. A total of 160 Belgian Facebook profiles were analyzed. These profiles were not retained in any form, and identifying personal information was not used. | A codebook was developed based on a test sample of profiles. The definitive sample was content analyzed by the second author. Summarizing data on the level of the profile was collected. Next, every picture containing references to alcohol, as well as picture comments, was coded. Finally, information on the status updates that were made to alcohol use and the comments to these references were constructed by coding for the 20 last status updates. To determine interrater reliability, a random sample of 20 profiles was simultaneously coded by a second coder. All data were analyzed using SPSS 20.0. |
| Bender et al [37] | 2008 | 620 groups | Public | Breast cancer | Passive analysis | Performed a keyword search using the term breast cancer. A total of 620 public Facebook groups that met inclusion criteria were analyzed. Information such as general characteristics, membership, and user-generated content (including timeline posts) were extracted. | The first 100 groups’ content was analyzed to develop a coding and classification scheme to determine the general purpose of the groups. A second-tier coding scheme was developed to subclassify and describe the specific purpose of each group. In addition, a coding scheme was created to classify the age and geographic location of the creators of the support groups. Descriptive statistics were calculated using SPSS 17. |
| Bazarova et al [38] | 2011 | 6 most recent status updates, timeline posts, and private messages for 79 participants | Private | College students | Active analysis | Each participant copied their status updates, timeline posts, and private messages they had written into a Web survey. They also answered questions about their self-presentational concerns for every message and their familiarity with the target of each of the timeline posts and private messages. | A total of 1227 messages that met inclusion criteria were analyzed using the Mac version of Linguistic Inquiry and Word Count (LIWC) 2007. An additional category for *happy birthday* was added to the LIWC dictionary. Analyses were performed in SPSS. |
| Tower et al [39] | N/A | N/A | Private | Nurse education | Active analysis | A total of 198 second-year nursing students were invited to join a Facebook group via email. The invitation advised the group to post information related to their study. An academic initiated a discussion in the Facebook group. Subsequent posts were initiated. The text and associated attributes were downloaded onto a spreadsheet. | A conventional thematic analytic approach was used to analyze threads and corresponding posts. The process was inductive and involved using a 3-stage approach. Researchers immersed themselves in the data to obtain an overall sense of the data. Next, the researchers coded the data, creating categories and abstraction. Direct quotes were used to maintain rigor when displaying the findings. |

^a^Only information about Facebook methodologies were included in this table. For studies that described methodologies for Facebook and other social media sites, non-Facebook–related information was excluded.

^b^N/A: not available.
